# Finite Element Surface Registration Incorporating Curvature, Volume Preservation, and Statistical Model Information

**DOI:** 10.1155/2013/674273

**Published:** 2013-09-28

**Authors:** Thomas Albrecht, Andreas Dedner, Marcel Lüthi, Thomas Vetter

**Affiliations:** ^1^Department of Computer Science, University of Basel, Bernoullistrasse 16, 4056 Basel, Switzerland; ^2^Mathematics Institute, University of Warwick, Zeeman Building, Coventry CV4 7AL, UK

## Abstract

We present a novel method for nonrigid registration of 3D surfaces and images. The method can be used to register surfaces by means of their distance images, or to register medical images directly. It is formulated as a minimization problem of a sum of several terms representing the desired properties of a registration result: smoothness, volume preservation, matching of the surface, its curvature, and possible other feature images, as well as consistency with previous registration results of similar objects, represented by a statistical deformation model. While most of these concepts are already known, we present a coherent continuous formulation of these constraints, including the statistical deformation model. This continuous formulation renders the registration method independent of its discretization. The finite element discretization we present is, while independent of the registration functional, the second main contribution of this paper. The local discontinuous Galerkin method has not previously been used in image registration, and it provides an efficient and general framework to discretize each of the terms of our functional. Computational efficiency and modest memory consumption are achieved thanks to parallelization and locally adaptive mesh refinement. This allows for the first time the use of otherwise prohibitively large 3D statistical deformation models.

## 1. Introduction

Nonrigid registration remains one of the largest challenges in medical image analysis and computer vision. The correspondence it seeks to establish between related objects is essential for a large number of applications from shape statistics to model-based segmentation and computational anatomy [1, 2].

In medical applications, the objects that need to be brought into correspondence are usually organs which were captured with a medical imaging device, such as CT and MRI. In this paper, we use bones, skulls, and hands as examples. We propose a method to bring them into correspondence by registering a number of feature images. The unique features and contributions of our method are as follows:

1. the representation of surfaces by a distance and a curvature image,
2. the inclusion of a volume preservation term into the registration,
3. the introduction of prior knowledge in form of a statistical deformation model,
4. a continuous formulation of the registration method, including the deformation model, making the registration independent of its discretization,
5. an efficient finite element discretization based on the local discontinuous Galerkin method.

For us, as in many other applications, the ultimate goal is the construction of statistical shape models. Therefore, we are mostly interested in the shape of the organ's surface, which we represent by the two most prominent feature images in our method: a distance and a curvature image of the surface. Together, they provide a good description of the shape of an object. Other possible feature images, which are then simultaneously registered, encode additional information about the organs like the CT or MRI data. Registering all feature images together represents our assumption that a good registration result should bring all of these feature images into correspondence.

In addition, we need to incorporate prior knowledge about the registration result in order to be able to address the inherently ill-posed problem of image registration. The result is given in the form of a vector field, and in the most basic form of our registration method, we simply enforce the smoothness of this vector field by controlling the norm of its first derivative. However, it turns out that this regularizer, which is also found in the Demons or Diffusion registration algorithms [3, 4], allows large and unnatural looking volume change. By penalizing volume change, we impose our prior knowledge that the registration result should not compress or expand the objects excessively. This is achieved by penalizing the linearized volume change caused by the vector field.

While this generic knowledge about registration and the resulting regularization applies to most registration tasks, we can go even further and include specific prior knowledge about the objects under consideration. This is done by penalizing deformations that deviate from the space of known deformations for the specific type of object. This space is modeled by a statistical deformation model derived from previous registration results of the same object class, that is, the same organ.

One of the main contributions of this paper is the formulation, discretization, and minimization of the registration problem as a continuous functional, integrating all the different terms described above into a single analytic formula for the deformation field. This formulation allows for the simple enhancement of the scheme through further terms and for a straightforward discretization. In this paper, we present a memory-efficient and flexible schemes using adaptive finite elements; this approach is presented in a general setting. The results shown in this paper are obtained using the *local discontinuous Galerkin* method. This approach leads to a very simple formulation even of complex regularization terms and is well suited for the use with distributed grids and nonconforming adaptation. The implementation is based on DUNE-FEM [5], which uses the generic grid interface from the Dune library [6, 7]. We use the ALUGrid [8] which supports nonconforming local adaptivity and the possibility of domain decomposition and dynamic load balancing for parallel computations. These advances in efficiency and memory consumption enable us to perform registrations that where previously not possible. In particular the use of large 3D statistical deformation models, which would typically require in excess of 700 MB for each mode of variation with a uniform discretization, becomes possible for the first time. Pre- and postprocessing are performed using ITK and VTK [9, 10].

### 1.1. Prior Work

 Nonrigid registration is an extremely well researched problem. For an overview of registration methods we refer to the recent survey papers by Zitová and Flusser [11] (image registration), Audette et al. [12] (surface registration), and in particular the book by Modersitzki [4] for a thorough discussion of variational methods for image registration. The most basic form of our method, that is, leaving out all the optional terms, is closely related to Thirion's Demons algorithm [3] and Modersitzki's diffusion registration algorithm [4].

The idea of surface registration using a distance or level-set representation of surfaces has been introduced by Paragios et al. [13], and the inclusion of additional feature images, especially for parametrized surfaces, is used for instance in [14]. The use of curvature images has been presented in our previous paper [15].

Volume preserving image registration was introduced by Rohlfing et al. in [16] and Haber and Modersitzki in [17]. Rohlfing et al. include a term penalizing volume change in a B-spline-based registration framework, while Haber and Modersitzki enforce strict nonlinear volume preservation in a variational formulation. In our approach, we wish to allow a limited amount of volume change and therefore use a soft constraint, that is, an additive penalty term. For efficiency, we penalize only the linear part of the volume change, and we show that this is equivalent to the linear elastic regularization term first introduced by Broit [18] and Christensen et al. in [19], even though our motivation for using this regularizer does not stem from modelling the organs as elastic bodies.

The concept of statistical deformation models and their inclusion into registration algorithms has been researched by several groups [20–23]. However, these methods either constrain the registration result strictly to the span of the model or use an interlaced algorithm performing the statistical regularization in a separate step, whereas we present an integrated formulation, which builds on our previous publication [24]. Furthermore, all of the previous methods are formulated within the discretization framework preferred by the respective group, whereas our method provides a continuous formulation of the deformation prior and, independently, a finite element discretization of this prior.

The use of finite elements for image registration goes back at least as far as [25], and we published a first finite element registration algorithm in [15]. The final model derived in this paper results in an elliptic problem with a nonlinear forcing term. The finite element discretization for general elliptic problems has now been employed for decades and can be considered standard. A summary of the standard approach of conforming, continuous finite elements can be found in [26]. Since we are using nonconform locally adapted grids with distributed memory parallelization, we employ a discontinuous finite element approach. An overview of this class of schemes can be found in [27]. The method we use is based on the *local discontinuous Galerkin* scheme introduced in [28]. To compute the second order derivatives in the elliptic regularization operator, the model is rewritten as a system of first-order equations, and these terms are discretized element-wise using intercell fluxes to enforce consistency with the analytical model. This results in a small discretization stencil, which leads to a simple implementation on nonconform distributed grid structures. The flexibility of this approach leads to a very simple formulation in spite of the rather complex elastic regularization terms. Also, the freedom in the choice of the basis functions allows us to use an orthonormal basis resulting in a diagonal mass matrix which simplifies the statistical regularization term considerably.

## 2. Registration Method

In the following, we describe our registration method. At its core, it is an image registration method and as such can be used directly on images. But as our focus lies in registering the shape or surface of organs, we describe how the method can be used to register two surfaces Γ_0_, Γ_1_ ⊂ ℝ^*d*^. The surfaces can be segmented from medical images or acquired otherwise. We assume that they are already rigidly preregistered, for instance by Procrustes alignment [29], so that our algorithm only needs to recover the nonrigid component of the registration. Moreover, the preregistration enables us to choose a common rectangular image domain *Ω* ⊂ ℝ^*d*^ which contains both surfaces, so that we can represent each surface Γ by its signed distance function *I* : *Ω* → ℝ:

(1) $$\begin{array}{c} I\left(x\right)∶=\{\begin{array}{cc} \mathrm{dist}\left(x,\Gamma \right) & x\in \text{outside}\left(\Gamma \right)\\ 0 & x\in \Gamma \\ -\mathrm{dist}\left(x,\Gamma \right) & x\in \text{inside}\left(\Gamma \right), \end{array} \end{array}$$

where dist⁡(*x*, Γ) is the Euclidean distance from *x* to Γ and the inside and outside of Γ have to be assigned in a meaningful way. For open surfaces, for which inside and outside cannot be defined, an unsigned distance function can be used.

The aim of a registration algorithm is to find a deformation field *u* : *Ω* → ℝ^*d*^ such that the target surface's distance function *I*_1_ warped with this deformation field, that is, *I*_1_(*x* + *u*(*x*)), is as close as possible to the distance function of the reference surface given by *I*_0_. The registration result of the distance functions implies a registration of the surfaces.

We formulate the registration problem as a minimization problem. It is shown in [4] that virtually all registration methods can be interpreted in this way. The deformation field *u* is sought as the minimum of a functional which is the sum of two terms: *distance* and *regularization* terms. Thus, the registration problem consists of finding the minimum of the functional

(2) $$\begin{array}{c} 𝒥\left[u\right]=𝒟\left[u\right]+ℛ\left[u\right], \end{array}$$

with distance term *𝒟* and regularization term *ℛ*. The former measures the distance between the reference and the registration target. At its minimum, the warp of the target is as close as possible to the reference image. The regularization term on the other hand measures the smoothness or regularity of the registration result *u*. The smaller it is, the more regular the solution will be. By minimizing both terms simultaneously, we try to bring the reference and target as close together as possible while keeping the deformation field reasonably smooth. We believe that there is no single generic distance or regularization term that guarantees a good registration in every scenario. The notion of correspondence is application specific, and the more knowledge about the registration task at hand we can include into the method, the higher the chances will be to obtain a result that meets our requirements. For example, the most basic regularization term we propose simply penalizes the squared *L* ^2^-norm of the gradient. By including additional regularization terms, we can also penalize volume change or the deviation from a statistical model of the object to be registered, thus improving the registration result. The same holds for the distance measure, where the simplest approach measures the *L* ^2^ difference between the two surfaces. Again considerably better results can be achieved by including additional terms, for example, the curvature of the surfaces.

### 2.1. Distance Term

 The basis for the distance term *𝒟* is the *L* ^2^ difference between the warp of the signed distance images *I*_1_ and the reference *I*_0_ of the two surfaces to be registered:

(3) ||I1(x+u(x))−I0(x)||L2(Ω)2 ∶=∫Ω(I1(x+u(x))−I0(x))2dx.

The distance images of two similar surfaces have a similar range of values, especially close to the surfaces, which makes the *L* ^2^ distance measure an appropriate choice for their comparison. In order to prevent undesirable effects at the boundary, where the distance function of each surface may be cut off at a different value, we bound the distance images at a certain distance *b* from the surface:

(4) $$\begin{array}{c} I^{b}\left(x\right)∶=\{\begin{array}{cc} I\left(x\right) & \text{if}\;\;I\left(x\right)\leq b\\ b & \text{if}\;\;I\left(x\right)>b, \end{array} \end{array}$$

and register these bounded distance images instead of the original *I*(*x*). The bound *b* ∈ ℝ should be chosen so that the *b* level set of each surface we want to register is completely contained inside *Ω*.

### 2.2. Robust Distance Measures

For noisy or otherwise difficult feature images it can be advantageous to use a robust distance measure, which dampens the influence of the overly large differences between the images; see [30] for a review of different robust cost functions. We propose using a robust distance measure based on the Geman-McClure estimator [30, 31], which has been successfully used for medical image registration in [32]. It can be easily realized by weighting the distance measure (3) with a term *Q*_*I*_(*x*):

(5) $$\begin{array}{c} {𝒟}_{I}\left[u\right]∶=\frac{1}{2}\int_{\Omega }\frac{1}{Q_{I}\left(x\right)}\;{\left(I_{1}\left(x+u\left(x\right)\right)-I_{0}\left(x\right)\right)}^{2}dx. \end{array}$$

For the Geman-McClure distance measure, we have *Q*_*I*_(*x*) = *C* ^2^ + (*I*_1_(*x* + *u*(*x*)) − *I*_0_(*x*))^2^, with a regularization parameter *C* ∈ ℝ which controls the robustness of the measure. A similar term is used in Thirion's Demons algorithm [3], where the norm of the gradient of the image replaces *C*: *Q*_*I*_(*x*) = |∇*I*_1_(*x*+*u*(*x*))|^2^ + (*I*_1_(*x* + *u*(*x*)) − *I*_0_(*x*))^2^. In our experiments, both weights yielded similar results, which is not surprising as for distance functions |∇*I*| = 1 almost everywhere. We have found that for distance images that represent surfaces free from artifacts or excessive noise, it is not necessary to use a robust distance measure. But it proved to be of good use for the additional feature images introduced in the following sections, such as the curvature images.

#### 2.2.1. Curvature Guided Registration

 When registering surfaces by means of their distance images, the problem arises that by definition, and the value of the distance function is zero on the whole surface and therefore contains no information on the surface. Therefore, the distance function *𝒟* is minimized whenever a point on one surface is registered onto a point on the other surface even if it does not correspond functionally or semantically to the given point. In fact, when we try to minimize the registration functional with a gradient descent scheme, the corresponding point is only sought in the direction of the gradient of the distance image, that is, perpendicular to the surface. This effect is somewhat alleviated by the regularization term, but this is often not enough to obtain a sensible registration. See the example in Section 4.3 for instance.

**Figure 1:**
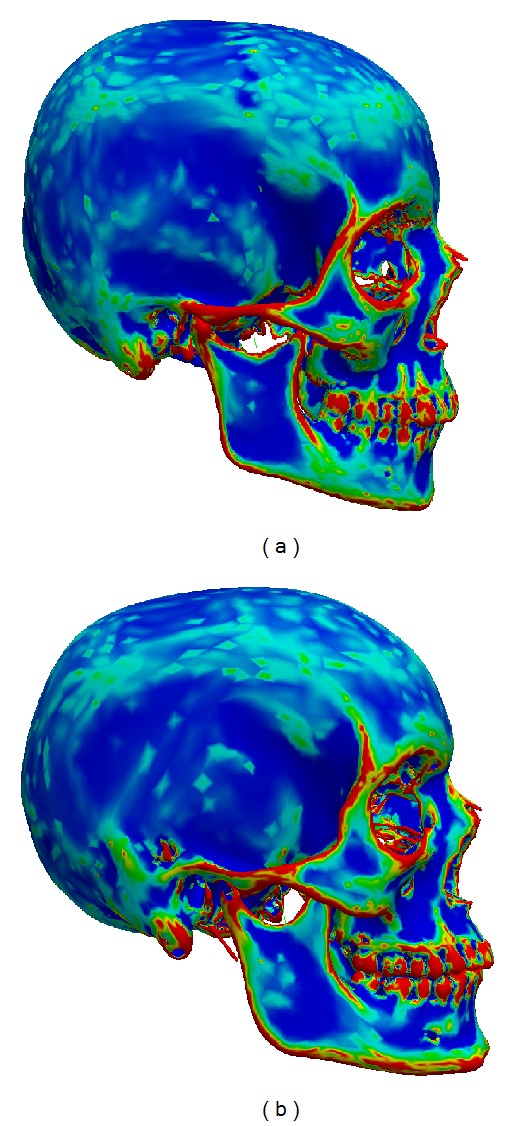
Two skulls colored according to their mean curvature. We see that corresponding points have similar mean curvature.

For bone registration, we wish to establish correspondence between points that have a similar anatomical function. So similar bumps, crests, ridges, and so forth should be matched. Such features are well described by the curvature of the surface. In fact, for a large class of objects, corresponding points on two surfaces have similar curvature.  Figure 1 illustrates this for the mean curvature of human skulls. Therefore, we use the mean curvature as an additional feature to be matched.

With the surfaces represented by their distance images, the curvature is easily calculated by *H*(*x*) = div⁡(∇*I*/||∇*I*||). For each *x* ∈ *Ω*, *H*(*x*) is the mean curvature of the level surface passing through *x*. So if *x* is on the zero level set of *I*, *H*(*x*) is the mean curvature of the surface at that point. Since for distance images ||∇*I*|| = 1 almost everywhere, the curvature image *H* is even more easily computed as *H* = Δ*I*.

The curvature images are included in the registration process with a distance term analogous to that in (5) as follows:

(6) $$\begin{array}{c} {𝒟}_{H}\left[u\right]∶=\frac{1}{2}\overset{}{\underset{\Omega }{\int }}\frac{1}{Q_{H}\left(x\right)}{\left(H_{1}\left(x+u\left(x\right)\right)-H_{0}\left(x\right)\right)}^{2}dx. \end{array}$$

The overall distance measure is then given as *α𝒟*_*I*_[*u*] + *β𝒟*_*H*_[*u*] with *α*, *β* ∈ ℝ^+^ controlling the influence of the distance and curvature images.

#### 2.2.2. Additional Feature Images

 In an obvious fashion, any number of additional feature images can be added. If we denote the *k*th pair of feature images by *X*_0_^*k*^, *X*_1_^*k*^, the full distance term for our registration method is given as

**Figure 2:**
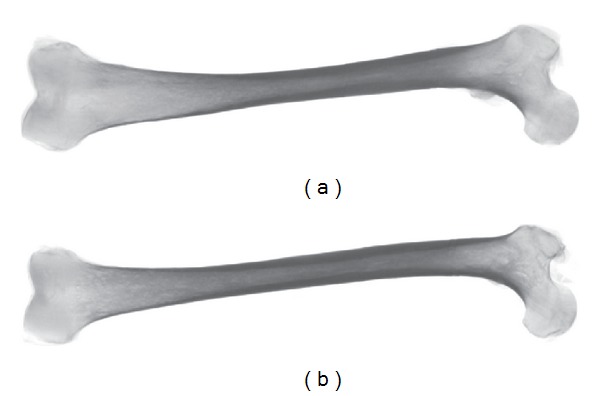
2D projections of CT scans of two femurs.

(7) 𝒟[u]∶=∑k=1nαk  𝒟Xk[u]=∑k=1nαk2∫Ω1QXk(X1k(x+u(x))−X0k(x))2dx,

with weighting parameters *α*_*k*_. In our experiments, we have included the original CT scans from which the bone surfaces were segmented as additional feature images where they were available. 2D projections of two such CT scans can be seen in Figure 2. Additional image modalities or manual annotations could also be included provided that they are available for both surfaces to be registered. Obviously for each of the surfaces, all its feature images need to be in correspondence, which is usually already the case or can be achieved with a rigid registration method. For multimodal image pairs, more sophisticated multimodal distance measures can be employed instead of the *L* ^2^ distance measures used in (7) (cf. [4, 33]).

### 2.3. Regularization Term

 Registration is an ill-posed problem, and any algorithm trying to minimize a distance measure without enforcing some kind of smoothness or regularity on the solution is bound to fail. We begin by introducing a very basic regularization term, which is later enhanced by adding further terms.

One of the most basic ways to control the smoothness of the deformation field *u* is through its first derivative *Du*, which we will, for simplicity, denote by ∇*u*. But the reader should bear in mind that *u* : *Ω* ⊂ ℝ^*d*^ → ℝ^*d*^, and hence ∇*u* ∈ ℝ^*d*×*d*^. We define the basic regularization term as

(8) ℛg[u]∶=12∫Ω||∇u||2dx=12∑l=1d∫Ω|∇ul|2dx.

The smaller *ℛ*_*g*_[*u*] is, the smoother the deformation field *u*.

#### 2.3.1. Volume Preservation

 While the regularization term (8) forces the deformation field to be smooth, it still allows some quite unnatural deformations. In particular, it allows for excessive expansion or compression of the registered object by the deformation field; see Section 4.4 for an example. The compression or expansion of the warp *x* + *u*(*x*) can be measured by the determinant of its first derivative det⁡[*D*(*x* + *u*(*x*))]. A volume preserving deformation field must satisfy det⁡[*D*(*x* + *u*(*x*))] ≡ 1. Naturally, as we are mostly interested in registering bones of different individuals, which in general do not have the same volume, we do not wish to enforce a strict incompressibility constraint as in fluid dynamics or other registration approaches [17]. Instead, we add a soft incompressibility constraint to our existing regularization term based on the linearization of det⁡[*D*(*x* + *u*(*x*))]. Thus, volume change is limited but not completely prohibited.

Since det⁡[*D*(*x* + *u*(*x*))] = 1 + div⁡*u* + (*nonlinear terms*), if follows that the smaller the divergence of *u*, the closer the linearization of the determinant is to 1 and therefore the field to being volume preserving, provided the values of *Du* are not too large and therefore the linearization justified. Therefore we add the square of the deformation field's divergence to the functional:

(9) ℛ[u]∶=μℛg[u]+νℛd[u]=μ12∫Ω||∇u||2dx+ν12∫Ω(div⁡u)2dx.

In a later section, we will see that the Euler-Lagrange equations for the functional (9) correspond to those of the well-known linear elastic registration methods (see Appendix A). Although the volume preservation is only approximative, it enhances the registration result visibly; see Section 4.4.

#### 2.3.2. Statistical Deformation Prior

It is well known that the regularization terms can be interpreted from two different perspectives. On the one hand, they serve as numerical stabilizers and make the numeric treatment of ill-posed problems feasible. On the other hand, the regularization term incorporates prior knowledge about the solution into the problem. In this respect, the regularization terms that we have introduced so far were generic in the sense that they only require the solution to be smooth or volume preserving. They do not take properties of the object to be registered into account. Even though registration is a prerequisite for expressing prior knowledge about a specific class of objects, it can itself benefit from such prior knowledge when the data is noisy. Therefore, we propose including prior knowledge about the specific class of objects by introducing an additional regularization term. This term is based on a probability distribution estimated from previous successful registration results and penalizes unlikely deformations. This type of regularization becomes very natural when we consider the probabilistic interpretation of the regularization approach. The regularization term *ℛ*[*u*] can be seen as defining a prior probability distribution *p*[*u*] over the function space, and the distance term *𝒟*[*u*] defines the likelihood *p*[*D* | *u*] of observing the data given deformation field *u*. See Wang and Staib [21] for a detailed discussion of this probabilistic interpretation for variational image registration.

Assume that we already have a set of *n* deformation fields {*u*_1_,…, *u*_*n*_}, mapping from a common reference image *I*_*R*_ to a given set of target images {*I*_1_,…, *I*_*n*_}. We expect that a new registration result is unlikely to be very different from the ones already given, since it registers an object of the same class. Indeed, we can regard the deformation fields *u*_1_,…, *u*_*n*_ as training data from which we can estimate a probability distribution over the possible deformations. This is the main idea behind *Statistical Shape Models* [34, 35] and the related *Statistical Deformation Models* [22], to which our problem belongs. In these approaches, the common assumption is that the objects (i.e., the shapes and the deformation fields) follow a normal distribution. Its parameters are estimated from the training data.

We introduce a continuous formulation for deformation models here, which fits naturally into our continuously defined registration framework and permits a straight-forward finite element discretization. The model is characterized by the mean field $\bar{u}$

(10) $$\begin{array}{c} \bar{u}\left(x\right)=\frac{1}{n}\overset{n}{\underset{i=1}{\sum }}u_{i}\left(x\right) \end{array}$$

and the covariance operator *𝒞* : *L* ^2^(*Ω*, ℝ^*d*^) → *L* ^2^(*Ω*, ℝ^*d*^)

(11) $$\begin{array}{c} 𝒞=\frac{1}{n}\overset{n}{\underset{i=1}{\sum }}\left(u_{i}-\bar{u}\right)⊗\left(u_{i}-\bar{u}\right), \end{array}$$

which acts on a deformation field *w* ∈ *L* ^2^(*Ω*, ℝ^*d*^) through

(12) 𝒞[w](x)=1n∑i=1n(ui−u¯)(x)   ×∫Ω(ui−u¯)(y)·w(y)dy.

This operator is the continuous analogon to the sample covariance matrix. It is a linear integral operator with a symmetric integral kernel given by

(13) $$\begin{array}{c} C\left(x,y\right)=\frac{1}{n}\overset{n}{\underset{i=1}{\sum }}\left(u_{i}-\bar{u}\right)\left(x\right){\left(u_{i}-\bar{u}\right)}^{T}\left(y\right). \end{array}$$

*𝒞* is a self-adjoint (i.e., symmetric) compact operator. Thanks to the spectral theorem for compact normal operators (see [36] for instance) it admits an eigenvalue decomposition with positive eigenvalues *σ*_*i*_^2^ and corresponding orthonormal eigenfunctions *ρ*_*i*_. As *𝒞* is estimated from *n* examples $u_{i}-\bar{u}$, there are at most *m* ≤ *n* nonzero eigenvalues; *σ*_*i*_^2^ and *𝒞* can be represented as

(14) $$\begin{array}{c} 𝒞\left[w\right]\left(x\right)=\overset{m}{\underset{i=1}{\sum }}\sigma_{i}^{2}\rho_{i}\left(x\right)\int_{\Omega }\rho_{i}\left(y\right)\cdot w\left(y\right)dy. \end{array}$$

Moreover, the spectral theorem guarantees the orthogonal decomposition of *L* ^2^(*Ω*, ℝ^*d*^) into the span of the eigenfunctions *ρ*_*i*_ and the operator's null space N(*𝒞*):

(15) $$\begin{array}{c} L^{2}\left(\Omega ,R^{d}\right)=\mathrm{span}\left\{\rho_{i}\;|\;i=1,\ldots ,m\right\}⊥\text{N}\left(𝒞\right). \end{array}$$

The span of the eigenfunctions is the same as the span of the $u_{i}-\bar{u}$. That means that it contains all linear combinations of the training examples. On this *m*-dimensional space, which is frequently called the *model space* of the deformation model, the covariance operator *𝒞* is invertible. Thanks to the eigenvalue decomposition, the inversion of *𝒞* on the model space is easily achieved by inverting the eigenvalues and coincides with the formulation of the pseudoinverse *𝒞* ^†^ on the whole space *L* ^2^(*Ω*) as follows:

(16) $$\begin{array}{c} {𝒞}^{†}\left[w\right]\left(x\right)=\overset{m}{\underset{i=1}{\sum }}\sigma_{i}^{-2}\rho_{i}\left(x\right)\overset{}{\underset{\Omega }{\int }}\rho_{i}\left(y\right)\cdot w\left(y\right)dy. \end{array}$$

We can therefore formally define a normal distribution $𝒩(\bar{u},𝒞)$ with mean $\bar{u}$ and covariance *𝒞* on *L* ^2^(*Ω*):

(17) $$\begin{array}{c} p_{1}\left(u\right)=\frac{1}{Z}e^{-(1/2){\langle u-\bar{u},{𝒞}^{†}[u-\bar{u}]\rangle }_{L^{2}(\Omega ,{\mathbb{R}}^{d})}}, \end{array}$$

where *Z* is a normalization parameter. Technically, while this function exists and is the natural extension of the density function of a statistical shape or deformation model to a continuous function space, it is not a well-defined density function. However, for any finite discretization *u*_*h*_, the corresponding density of the multivariate normal *𝒞*[*u*_*h*_] exists and is well defined. This distribution can be used as the basis for a statistical regularization term. However, it only represents prior knowledge about the model space. Its complement, N(*𝒞*), is completely ignored. A statistical regularization based on this distribution would permit deformation fields *u* ∈ *L* ^2^(*Ω*, ℝ^*d*^) that are arbitrarily far away from the model space and would regularize only their projection onto the model. An extreme measure taken by other authors is therefore to restrict the registration results strictly to the model space [20, 22]. We on the other hand wish to explicitly allow results that lie outside (but still close to) the model space, as only these results can be used as reasonable additions to the statistical model.

We define an additional distribution on N(*𝒞*), which imposes our prior knowledge that the registration results should not lie far from the model. In the absence of additional example data, we simply assume a normal distribution with mean $\bar{u}$ and uncorrelated uniform variance of *σ* ^2^ ∈ ℝ^+^, which controls how much the results are allowed to deviate from the model space:

(18) $$\begin{array}{c} p_{2}\left(u\right)=\frac{1}{Z}e^{-(1/2)\sigma^{-2}{\langle u-\bar{u},u-\bar{u}\rangle }_{L^{2}(\Omega ,{\mathbb{R}}^{d})}}. \end{array}$$

While this term could in principle be restricted to N(*𝒞*), we define it on the whole space *L* ^2^(*Ω*, ℝ^*d*^). This corresponds to shrinkage estimation, a technique used in statistics to improve the estimation of the covariance [24, 37], which adds the additional variance of *σ* also on the model space.

To define the distribution *p*(*u*) characterizing our complete statistical model, we assume the two normal distributions to be independent and define *p*(*u*) as their product

(19) p(u)∶=p1(u)p2(u)=1Ze−(1/2)〈u−u¯,𝒞†[u−u¯]〉 ×1Ze−(1/2)σ−2〈u−u¯,u−u¯〉.

It is then natural to introduce the functional

(20) 𝒫[u]=12  γ∫Ω  (u−u¯)(x)·𝒞†[u−u¯](x)dx +12  γ∫Ωσ−2(u−u¯)(x)·(u−u¯)(x)dx∝−ln⁡p[u]

as an additional regularization term, which penalizes unlikely deformation fields. The constant *γ* is used as a weighting factor. We thus arrive at the following functional describing our registration model:

(21) $$\begin{array}{c} 𝒥\left[u\right]=𝒟\left[u\right]+ℛ\left[u\right]+𝒫\left[u\right]. \end{array}$$

## 3. Finite Element Discretization

 In this section, we describe the discretization and minimization of the functional (21) based on a finite element method and an adaptive multiresolution pseudo-time stepping approach. We start with the description of the spatial discretization focusing on the terms from the deformation prior. This finite element discretization of the continuously defined deformation prior is one of the most important contributions of this paper, and thanks to use of a memory-efficient adaptive finite element basis makes the use of large 3D statistical deformations possible for the first time. 

### 3.1. Space Discretization

 To find a minimizer of the functional (21), we wish to solve the weak form of the corresponding Euler-Lagrange equation: *J*′[*u*, *φ*] = 0 for all suitable test functions *φ*. The details of how the derivative *J*′[*u*, *φ*] = *𝒟*′[*u*, *φ*] + *ℛ*′[*u*, *φ*] + *𝒫*′[*u*, *φ*], which can be interpreted as a bilinear form, is computed can be found in Appendix A. The three terms, corresponding to the distance measure, regularization term, and the deformation prior, respectively, are given by

(22) 𝒟′[u,φ]=∑k=1n∫ΩαkQXk(x)(X1k(x+u(x))−X0k(x))∇X1k(x+u(x))φ(x)dx,

(23) $$\begin{array}{c} {ℛ}^{\prime }\left[u,\varphi \right]=\int_{\Omega }\mu \nabla u:\nabla \varphi +\nu \mathrm{div}u\mathrm{div}\varphi dx, \end{array}$$

(24) 𝒫′[u,φ]=∫Ωγ  𝒞†[u−u¯](x)·φ(x)dx +∫Ωγ  σ−2(u−u¯)(x)·φ(x)dx.

The sum in (22) is over all feature images *X*_*k*_ used for the registration, for example, the distance maps *I*, the curvature *H*, and the CT scans.

We employ a finite element discretization to compute the deformation field. Given a function space *V*_*h*_ ⊂ *L* ^2^(*Ω*, ℝ^3^) of finite dimension *N*, we seek an approximate deformation field, which we will also denote by *u* ∈ *V*_*h*_, satisfying *𝒥*′[*u*, *φ*] = 0 for all test function *φ* ∈ *V*_*h*_.

After choosing basis functions *φ*_1_,…, *φ*_*N*_ of *V*_*h*_, a function *w* ∈ *V*_*h*_ can be represented as *w*(*x*) = ∑_*k*=1_^*N*^w_*k*_ *φ*_*k*_(*x*) with a vector of degrees of freedom (DOF) denoted by **w**_*h*_ = (w_*k*_)_*k*=1_^*N*^.

The derivation of a discrete version both for the regularization term *ℛ*′ and the distance measure *𝒟*′ is straightforward and will be briefly sketched in Section 4.1 together with details on the construction of the discrete function space *V*_*h*_ used in our tests. In the following, we will concentrate on the term *𝒫*′ arising from the statistical deformation prior.

The term *𝒫*′ defined in (24) includes the eigenfunctions of the covariance operator *𝒞*, defined in (12). The eigenfunctions are needed for the efficient (pseudo-) inversion of *𝒞* according to (16). In order to formulate a discretization and an implementation for *𝒫*′, we need to actually calculate these eigenfunctions. According to the ideas of functional PCA [38], the eigenfunctions can be calculated with help of a finite dimensional eigenvalue decomposition if both the argument *w* and the training examples *u*_*i*_ are given as a linear combination of a set of basis functions.

So to derive a discrete formulation of *𝒞*, we fix the argument *w* and examples *u*_*i*_ to be from the discrete function space *V*_*h*_. They are then given as linear combinations of the basis functions *φ*_*k*_, and we have the coefficient vectors **w**_*h*_ and $v_{h,i}∶=u_{h,i}-{\bar{u}}_{h}$. The covariance operator *𝒞* from (12) then takes the form

(25) 𝒞[w](x)=1n∑i=1n∑kvikφk(x)∫Ω∑jvijφj·∑łwlφldy=∑k(1n∑i=1n∑l,jvikvij∫Ωφj·φl  dy  wl)φk(x)=∑k(Awh)kφk(x).

The matrix **A** = (*a*_*kl*_) is given by

(26) $$\begin{array}{c} a_{kl}=\frac{1}{n}\overset{n}{\underset{i=1}{\sum }}\overset{N}{\underset{j=1}{\sum }}{\text{v}}_{ik}{\text{v}}_{ij}m_{jl}, \end{array}$$

where we have used *m*_*jl*_ to denote the entries of the mass matrix **M**; that is, *m*_*jl*_ = ∫_*Ω*_ *φ*_*j*_(*y*) · *φ*_*l*_(*y*)*dy*.

Equations (12) and (26) show that *𝒞* is restricted to *V*_*h*_ maps to *V*_*h*_ and is represented by the matrix **A** = *a*_*kl*_, which, resubstituting the definition of v_*ik*_ into (26), can be represented as

(27) $$\begin{array}{c} A=\frac{1}{n}\overset{n}{\underset{i=1}{\sum }}\left(u_{hi}-{\bar{u}}_{h}\right){\left(u_{hi}-{\bar{u}}_{h}\right)}^{T}M=:\Sigma M, \end{array}$$

where Σ is the sample covariance matrix of the DOF vectors $v_{h,i}=u_{hi}-{\bar{u}}_{h}$. The eigenvalue decomposition of this discrete version of *𝒞* can be achieved by an eigenvalue decomposition of the matrix **A**. Compared with traditional discrete statistical models as [22, 34, 35], where only the covariance matrix Σ is used, in our case, the mass matrix **M** slightly complicates matters as it makes the matrix **A** nonsymmetric in general. In functional PCA, this problem is solved by first calculating an eigenvalue decomposition of the symmetric matrix:

(28) $$\begin{array}{c} M^{1/2}AM^{-1/2}=M^{1/2}\Sigma M^{1/2}. \end{array}$$

This is a symmetric positive semidefinite (*N* × *N*)-matrix with rank *m* ≤ *n* ≪ *N*. Remember that *n* is the number of training examples *u*_*i*_ which is usually much smaller than *N*, the number of degrees of freedom of the discrete function space *V*_*h*_. The positive eigenvalues *σ*_1_^2^,…, *σ*_*m*_^2^ and the matrix **S** of their corresponding orthonormal right eigenvectors **r**_*i*_ can be computed efficiently with a singular value decomposition of an (*m* × *m*) matrix, without the need to compute a full large (*N* × *N*) singular value decomposition. We thus have the eigenvalue decomposition:

(29) $$\begin{array}{c} M^{1/2}AM^{-1/2}=S\mathrm{diag}\left(\sigma_{1}^{2},\ldots ,\sigma_{m}^{2}\right)S^{\text{T}} \end{array}$$

and consequently the decomposition of **A**:

(30) $$\begin{array}{c} A=M^{-1/2}S\mathrm{diag}\left(\sigma_{1}^{2},\ldots ,\sigma_{m}^{2}\right)S^{\text{T}}M^{1/2} \end{array}$$

with eigenvalues *σ*_*i*_^2^ and eigenvectors **M** ^−1/2^ **r**_*i*_. The associated functions *ρ*_*i*_ = ∑_*k*_(**M** ^−1/2^ **r**_*i*_)_*k*_ *φ*_*k*_ are the eigenfunctions of the discretized covariance operator from (25). *𝒞* being a symmetric operator, we expect its eigenfunctions to be orthonormal with respect to the *L* ^2^(*Ω*, ℝ^3^) scalar product:

(31) $$\begin{array}{c} \int_{\Omega }\rho_{i}\cdot \rho_{j}=r_{i}^{T}M^{-1/2}MM^{-1/2}r_{j}=r_{i}^{T}r_{j}=\delta_{ij}. \end{array}$$

With the eigenfunctions *ρ*_*i*_, we can rewrite (24) with an explicit formulation for the pseudoinverse *𝒞* ^†^ as follows:

(32) 𝒫′[u,φ]=∑i=1mγ  σi−2∫Ω(u−u¯)(y)·ρi(y)dy   ×∫Ωρi(x)·φ(x)dx +γσ−2∫Ω(u−u¯)(x)·φ(x)dx,

which can easily be implemented once the eigenfunctions *ρ*_*i*_ have been calculated.

This formulation takes the same form in the continuous, and the discrete setting and could have been calculated directly using the continuous eigenvalue decomposition Equation (16). The discretization lies only in the restriction of *𝒫*′ to the discrete function space *V*_*h*_ and the calculation of the eigenfunctions via a matrix SVD as described above.

### 3.2. Minimization Strategy

 Registration is achieved by minimizing the functional (21). We introduce an artificial time variable *t* and try to minimize the functional by computing its gradient flow, that is, we solve the time-dependent partial differential equation:

(33) $$\begin{array}{c} \partial_{t}u-{𝒥}^{\prime }\left[u\right]=0 \end{array}$$

for a function *u*(*x*, *t*), given an initial solution *u* ^0^ = *u*(*x*, 0). Note that the term ∂_*t*_ *u* has to be interpreted in the weak sense, that is, in the form ∫_*Ω*_∂_*t*_ *uφ*. A time stepping scheme for (33) can be viewed as a continuous version of gradient descent; indeed, if we use an Euler scheme for the time discretization of (33), we end up with a gradient descent iteration.

Choosing an explicit Euler scheme to solve (33) leads to a severe time step restriction, coupling the time step *τ* with the mesh width *h* via *τ* = *O*(*h* ^2^) due to the regularization term (8). On the other hand, due to the nonlinearity of the distance term *𝒟*[*u*], a purely implicit iteration scheme would require the solution of a large nonlinear system in each iteration. Therefore, we propose to use semi-implicit time stepping scheme. We split up the functional *𝒥*[*u*] into an explicit part *𝒥*_expl_[*u*], containing all terms with nonlinear derivatives and an implicit part *𝒥*_impl_[*u*] containing all terms with linear derivatives. The iteration scheme, in its simplest form, is then defined as

(34) $$\begin{array}{c} u^{n+1}=u^{n}+\tau {𝒥}_{\text{impl}}^{\prime }\left[u^{n+1}\right]+\tau {𝒥}_{\text{expl}}^{\prime }\left[u^{n}\right]. \end{array}$$

In our case, this means that *𝒥*_expl_ = *𝒟* and *𝒥*_impl_ = *ℛ* + *𝒫*. If additional terms are introduced, they can be added to either *𝒥*_expl_ or *𝒥*_impl_, depending on their linearity. Each iteration step thus requires the solution of a *linear* system of equations for which we employ an iterative Krylov type solver (in this case the BiCGStab method). To obtain higher order version of this method, IMEX Runge-Kutta schemes are used [39].

**Figure 3:**
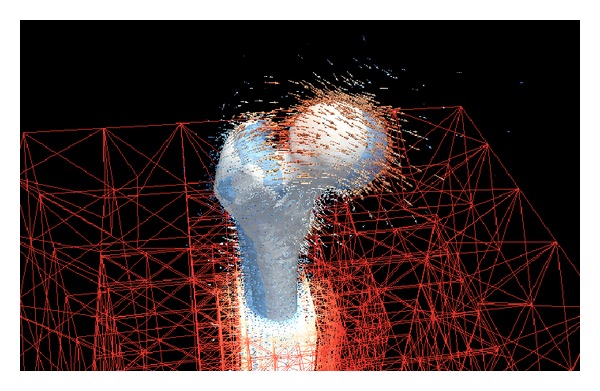
Visualization of the registration of two femurs. The deformation field that deforms one femur into the other is calculated on an adaptive grid.

For our experiments, we chose a locally adaptive multiresolution strategy; we first minimize the functional on a coarse uniform grid; that is, starting with the initial guess *u* ^0^ ≡ 0, we solve (33) up to some fixed time *T*_0_. Then the grid is refined around the reference surface, and the coarse solution is interpolated onto the refined grid. This is then used as starting value for solving (33) on the refined grid up to some fixed time *T*_1_. This process is repeated until a sufficiently fine resolution has been reached. The times *T*_*i*_ have been experimentally determined. In each step, all elements closer to the surface than a certain threshold Θ are refined; that is, |*I*_0_ | <Θ. This threshold is decreased in each step, so that the final solution is calculated on a grid that offers a very fine resolution close to the reference surface and becomes more and more coarse further out. An example is shown in Figure 3.

## 4. Results

### 4.1. Implementational Details

 The scheme is implemented in the Dune framework, a software library allowing the generic implementation of grid based numerical schemes [6, 7]. The finite element implementation is based on the DUNE-FEM module [5]. Pre- and post-processing is done using ITK and VTK [9, 10].

The spatial discretization of the deformation field *u* is based on a tessellation *𝒯*_*h*_ = {*T*_*i*_}_*i*∈*ℐ*_ of the image domain *Ω*. This tessellation must be nonoverlapping and can be uniform or adaptive and is typically made up of triangles or rectangles in 2D or tetrahedra or hexahedra in 3D. We use the ALUGrid library [8] which supports unstructured meshes in 2D and 3D with nonconforming local adaptivity and the possibility of domain decomposition and dynamic load balancing for parallel computations. Figure 3 shows a visualization of such a nonconforming locally adaptive grid.

The spatial discretization employed is based on the *local discontinuous Galerkin* (LDG) scheme. Given a tessellation *𝒯*_*h*_, this scheme follows the same ideas as the standard Galerkin method [26] but employs a discontinuous ansatz space: *V*_*h*_^*q*^∶ = {*v*_*h*_:*v*_*i*_ ∈ [*P*_*q*_(*T*_*i*_)]^*d*^  for  *i* ∈ *ℐ*}. Here *v*_*i*_ ≡ *v*_*h*∣*T*_*i*__ and *P*_*q*_(*T*_*i*_) denote the space of polynomials on the element *T*_*i*_ of order *q*. Note that there is no continuity assumption between elements, so that there is very little restriction on the tessellation. Due to the discontinuous ansatz space, the discretization of the higher order derivatives in the regularization term is slightly more involved than in the standard Galerkin method. We briefly sketch the LDG approach when applied to a partial differential equation of the form $-\mu \Delta u-\nu \nabla \mathrm{div}u=\tilde{R}$, where $\tilde{R}$ combines all lower order terms. This equation corresponds to the strong form of the Euler-Lagrange equation (see Appendix A  ((A.9))). Rewriting the second-order terms as a first-order system for the vector valued functions *u*, *w*_1_,…, *w*_*d*_, we arrive at

(35) $$\begin{array}{c} w_{k}-\nabla u_{k}=0,\\ -\underset{i}{\sum }\partial_{i}\left(\mu w_{ki}+\nu w_{ik}\right)={\tilde{R}}_{k} \end{array}$$

with *k* = 1,…, *d*. This approach leads to a compact discrete form for the elastic regularization term *ℛ*′ involving only first-order derivatives on each element *T* ∈ *𝒯*_*h*_ and numerical fluxes over cell boundaries. Focusing on a single element *T* of the tessellation, the corresponding weak form of (35) takes the following form when we define the vector *W*_*k*_ : = (*μw*_*ki*_ + *νw*_*ik*_)_*i*_:

(36) $$\begin{array}{c} \int_{T}w_{k}\cdot \xi +\int_{T}u_{k}\nabla \cdot \xi -\int_{\partial T}\hat{u_{k}}\xi \cdot n=0\\ \int_{T}W_{k}\cdot \nabla \psi -\int_{\partial T}\hat{W_{k}}\psi =\int_{T}{\tilde{R}}_{k}\psi . \end{array}$$

The fluxes $\hat{u_{k}}$ and $\hat{W_{k}}$ can for example be taken as averages of the values on both sides of the boundary of *T* or using suitable one-sided values. For more details on the LDG method see [27, 28]. At the boundary we use homogeneous Dirichlet conditions for the deformation field *u*. This is a reasonable assumption if we use bounded distance images as defined in (4). In this case, the images *I*_0_ and *I*_1_ are both equal to the constant bound *b* near the domain boundary, and the functional only contains the regularization term *ℛ* in this region; that is, only the regularization operator remains for which zero boundary conditions can be prescribed.

In our implementation, we use orthogonal basis functions {*φ*_*l*_}. These are easily constructed by choosing on each element *T* a polynomial basis (*ϕ*_*α*_^*T*^)_*α*=1_^*r*^ of [*P* ^*q*^(*T*)]^*d*^ satisfying

(37) $$\begin{array}{c} \int_{T}\phi_{\alpha }^{T}\cdot \phi_{\beta }^{T}=\left|T\right|\delta_{\alpha \beta }. \end{array}$$

A basis function *φ*_*l*_ of *V*_*h*_^*q*^ is then chosen to coincide with a polynomial basis function *ϕ*_*α*_^*T*^ on one element *T* and vanish on all other elements. Thus the mass matrix *M* is a diagonal matrix of the form

(38) $$\begin{array}{c} M=\mathrm{diag}\left(\left|T_{1}\right|I_{r},\ldots ,\left|T_{s}\right|I_{r}\right), \end{array}$$

where *I*_*r*_ is the identity matrix with *r* = dim⁡([*P* ^*q*^(ℝ^*d*^)]^*d*^) and *s* is the number of elements in the grid; that is, the total number of DOFs is *N* = *sr*. The diagonal form of *M* makes its storage and the calculation of the weighted sample covariance matrix Σ defined in (27) very efficient.

Thus, the LDG method used here not only allows us to efficiently use adaptivity and parallelization but also makes the computation of the PCA simple and efficient with respect to memory consumption and computational cost.

### 4.2. 3D Surface Registration

**Figure 4:**
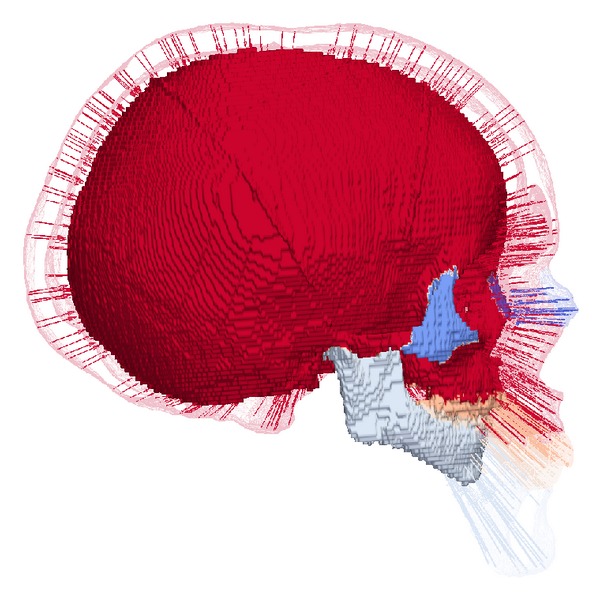
Visualization of the correspondence and the transfer of anatomical labeling between the reference (transparent outline) and a child's skull.

In this first experiment, we can observe that thanks to the level set representation of the surfaces, our method allows the accurate registration of surfaces. See Figure 3 for an example of the femur bone and Figure 4 for an example with the more complicated surface of the human skull, where our method benefits from the fact that the level set representation is independent of the topology of the surface. The topology of the skull surface is very complicated and, due to acquisition and segmentation artifacts, not necessarily the same for two different segmented skull surfaces.

**Figure 5:**
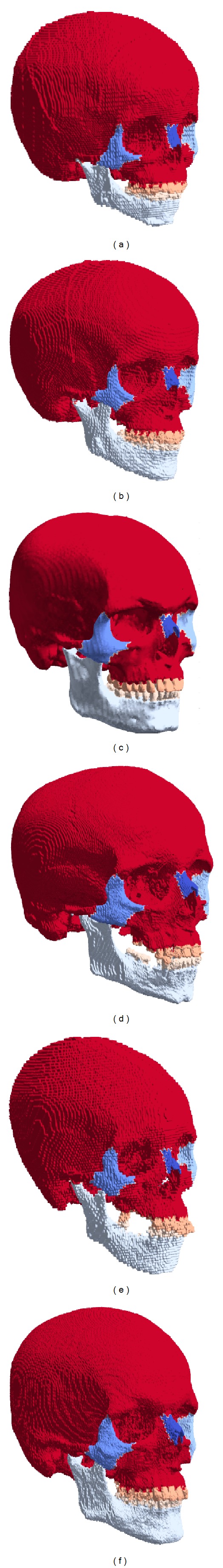
Transfer of the anatomical labelling of the reference (c) to a variety of skulls. Such a transfer requires very accurate registration results. Even a mismatch in the number of teeth is handled properly.

In our experiments, only the reference was anatomically labeled and hand segmented with high attention to detail. Figure 4 visualizes how this labelling and segmentation is transferred to another skull anatomy via the registration result.  Figure 5 shows that this works over a very large range of examples from infant skulls to large adult skulls. Even though the concept of correspondence breaks down for different numbers of teeth in the reference and the target, the existing teeth are labelled correctly.

On a standard 3 GHz dual-core desktop PC with two parallel registration processes, a skull registration with 480 000 degrees of freedom (i.e., 160 000 grid points) takes about 10 minutes, a femur registration with 80 000 degrees of freedom (but more iterations) about 6 minutes. These are only indicative times to give the reader a feeling for the run time of our algorithm. Computation times can be further reduced with more parallel processes and more aggressive parameter tuning. Note that a great advantage of our method is that the adaptive discretization requires a much inferior number of degrees of freedom than a uniform discretization. A uniform discretization with a similar resolution around the surface requires about 18 million degrees of freedom, resulting in a memory consumption of over 700 MB per deformation field.

### 4.3. Curvature Term

The above experiments were performed with all of the terms introduced in Section 2 except the statistical prior. We will now report on a couple of experiments that visualize the benefit of all the additional terms, starting with the curvature term introduced in Section 2.2.1.

**Figure 6:**
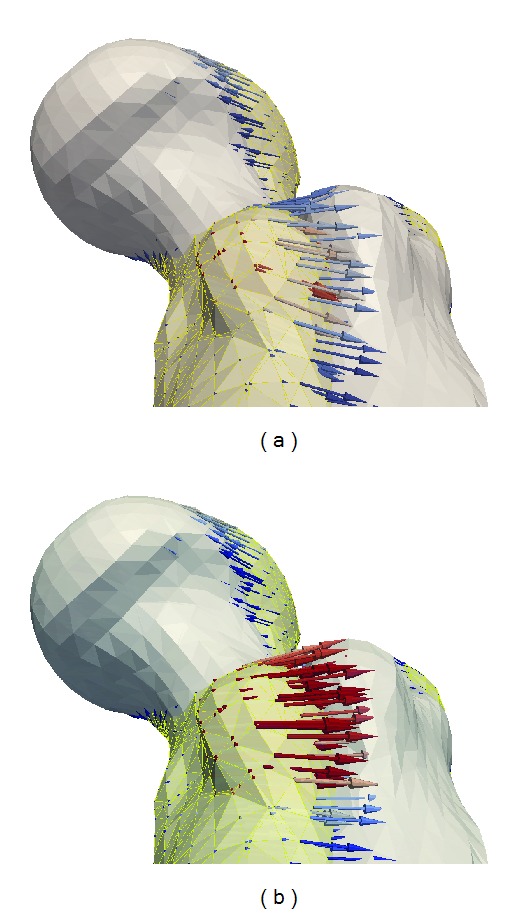
Registration of two femurs with and without curvature term. Without the curvature term, the correspondence is faulty. The corresponding features of the trochanter minor are not properly matched. The curvature term ensures matching of corresponding shape features.

In Figure 6(a), a registration of a femur is performed without the curvature term; that is, the surface is only represented by the level set function and the original CT scan. At a first glance, the surfaces are well registered, and the deformation field allows us to deform the reference bone so that it coincides with the target bone. However, on closer inspection, the implied correspondence is faulty. The deformation field matches the top of the trochanter minor (an important anatomical feature of the femur) of the reference to the side of the trochanter minor on the target. Such faulty correspondence causes problems in all subsequent applications such as building of statistical models or transferring anatomical labels.

In Figure 6(b), when the curvature term is used, the correspondence is much more sensible, and top is matched to top and sides to sides. The mean curvature proves to be a good description of anatomical features of bones. Its use in the registration method ensures superior correspondence.

### 4.4. Volume Preservation

**Figure 7:**
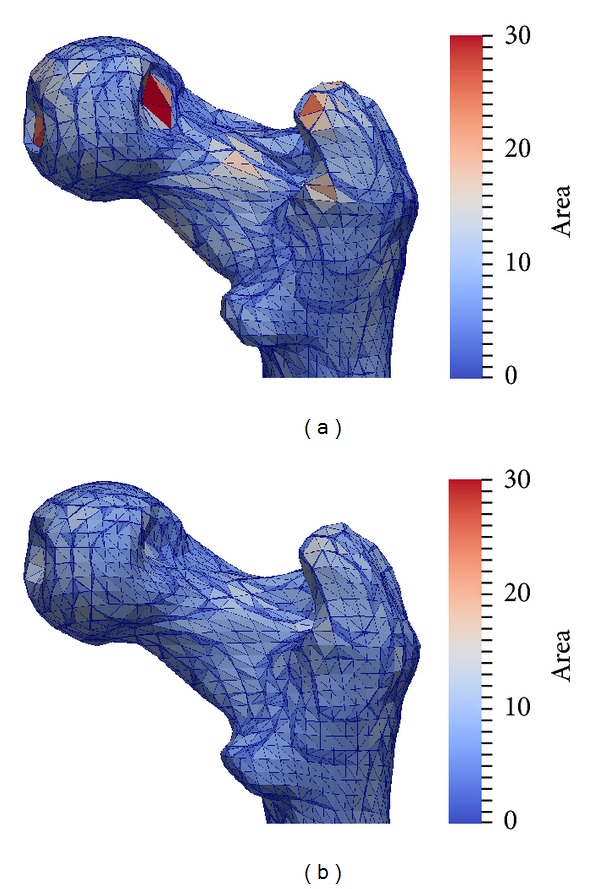
Registration of two femurs with and without volume preserving term. When the term is used, the distribution of area over the triangles of the mesh is much more even because the limited volume change prohibits strong expansion or compression of the mesh.

Figure 7(a) shows the reference bone warped with a deformation field of a registration result. To amplify the effect for visualization purposes, we have warped the reference bone with the deformation field multiplied by 2. The coloring represents the size of the triangles that make up the surface. We see that in some places the original reference grid is quite unnaturally stretched resulting in very large triangles. This is the effect of large volume expansion in the deformation field. When this volume change is penalized with the volume preservation term, the resulting mesh is much more even, while still allowing an equally good matching of the target surface.

In Figure 7(a), the weighting parameters *μ* and *ν* from (8) have been chosen as *μ* = 2, *ν* = 0, that is, no volume preservation term. In Figure 7(b), as *μ* = *ν* = 2, that is, equal weight of the gradient and the divergence term. Simply augmenting the weight of the gradient term, for example, *μ* = 4, *ν* = 0 also results in less volume change, but still more than in Figure 7(b), while simultaneously decreasing the matching accuracy.

### 4.5. Statistical Deformation Prior

**Figure 8:**
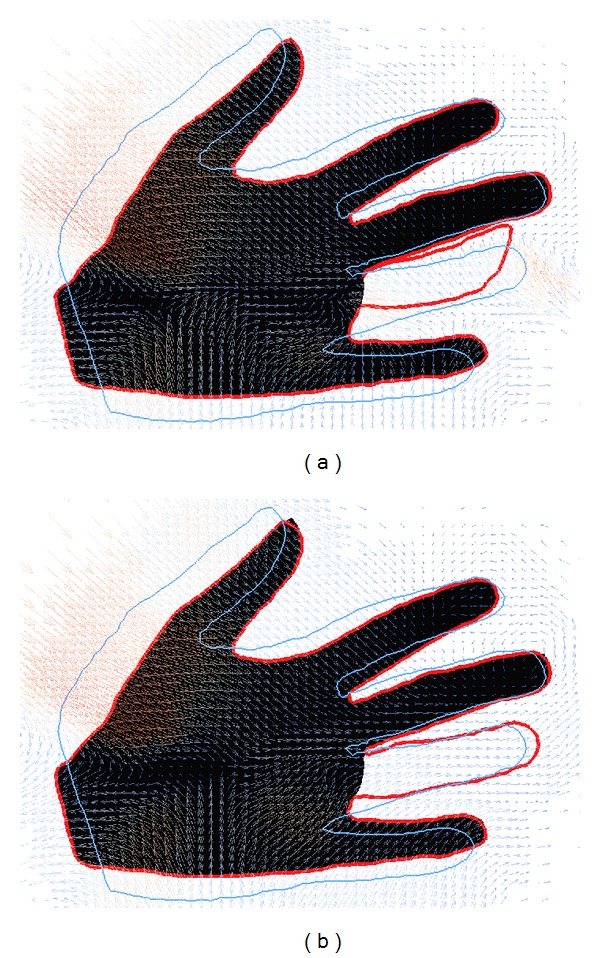
The reference shape (blue line) is registered onto a hand with a missing finger. The red line shows the warp of the reference with the resulting deformation field, without (a) and with statistical regularization (b).

In these final experiments, we exhibit the use and benefit of the statistical prior. We first performed registrations of 15 intact hands for the 2D example and 50 intact femurs for the 3D example. From these, statistical models are built according to the process described in Section 3.1. They can be used as prior knowledge for registering difficult or damaged data sets. In Figure 8, this prior knowledge allows the registration of a hand with a missing finger. Without this prior knowledge, the ring finger of the reference is deformed unnaturally in an attempt to match the hand with the missing finger. Note that the use of a robust distance measure and the regularization terms prevent a complete disappearance or distortion of the finger. When the prior knowledge from the statistical deformation model is used, the hand is well matched where intact. But the model enforces a registration with an intact ring finger, as all its training data sets include 5 intact fingers.

**Figure 9:**
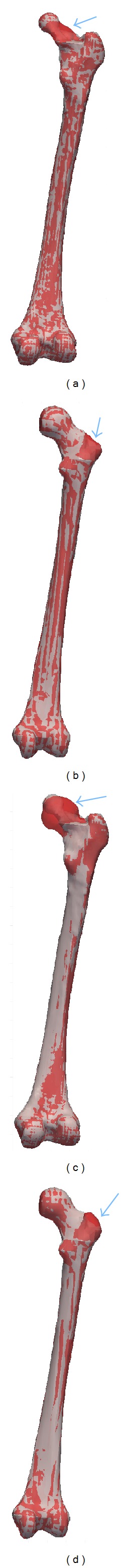
Registration of a pair of damaged bones, with and without statistical regularization. The first bone has an artificial hip joint, and in the second, the trochanter major is missing. Without statistical regularization, the damaged bones are matched exactly ((a), (b)). With statistical regularization, the method recognizes that the damaged parts do not conform with the prior knowledge and restore them automatically ((c), (d)).

In Figure 9, a similar experiment in 3D is shown. The prior knowledge from 50 previous registrations is used in the registration of the reference bone to two damaged bones. The first bone has an artificial hip joint prosthesis and the second one a missing trochanter major. The registration method with statistical regularization registers the complete bones while complementing the missing parts from its prior knowledge. In this way, we can see how the original bone could have looked like. In these experiments, we have used 40 modes of variation (principle components). In a uniform discretization with a similar resolution around the surface, this would have required 28 GB of memory. Of course, the inclusion of the statistical prior introduces additional computational complexity. This results in an increase of computation time from about 5 minutes without to about 7 minutes with statistical prior per femur registration.

These experiments were performed with a robust distance measure (cf.  Section 2.1). In the places with missing data, the regular *L* ^2^ measure has a very large value and would dominate the registration process. As with all regularization techniques, a balance between over- and under-regularization has to be found. If too much weight is placed on the statistical prior, the result is pulled too close to the model.

## 5. Discussion

We have presented a registration method which allows the accurate and efficient registration of surfaces by registering their distance images. While this can be achieved with any image registration algorithm, we have shown that the most obvious choices for the distance measure and regularizer have several shortcomings, which can be relieved by adding additional distance and regularization terms to the model. We have seen that by representing the surfaces not by their distance images alone but also by a combination of feature images, most notably the curvature images, and we can obtain superior correspondence information. Concerning the regularizer, we have shown that adding a volume preserving term results in more even and naturally looking deformations and warps. Finally, we have shown that including prior knowledge in form of a statistical deformation model allows us to register considerably damaged data sets. While the statistical prior could be used in any registration task where prior registration results are available, we have found that for intact data sets the method works well enough without the prior. We use the prior in cases where the data is too corrupted to be used directly, but where we still wish to add as much as possible of its information into our existing model.

Our registration method is formulated continuously and is independent of the discretization method. We have presented a finite element discretization based on the local discontinuous Galerkin method, which allows for local grid adaption and a straightforward implementation of all the terms of our functional. The local grid adaption and parallelization limit the computational complexity and memory consumption, enabling us to perform registrations that were not possible with previous methods. In particular the inclusion of large statistical deformation models was not possible with previous methods based on uniform discretization such as the Demons Algorithm. This will become more and more important as the resolution of medical images increases in the future. The flexibility of the continuous functional and the LDG discretization allows the easy integration of other concepts and terms that are being developed in the field of image registration every day. On the other hand, the ideas and terms introduced here can be used with other discretization schemes or existing registration methods.

## Appendix

### A. Derivatives

In the following, we give details on how to derive the Euler-Lagrange equations, which characterize the minimum *u* of the functional *𝒥* given in (21). Using the standard methods from the calculus of variations, we compute the Gateaux derivatives for each of the terms that make up our registration functional. This leads to the bilinear forms used in the finite element discretization in Section 3. We conclude this section with the derivation of the partial differential equations, that is, the strong form, which characterize the deformation field.

#### A.1. Distance Measure

As the individual terms of the combined distance measure (7) are all of the same form, it suffices to calculate the first variation of the distance measure with only one feature image *𝒟*_*I*_[*u*] defined in (5) as follows.

(A.1) 𝒟I′[u,φ] =ddε𝒟I′[u+εφ]|ε=0 =ddε12∫Ω1QI  (I1(·+u(·)+εφ(·))−I0(·))2dx|ε=0 =∫Ω1QI  (I1(·+u(·))−I0(·))∇I1(·+u(·))φ(·)dx.

The dependency of the weighting term *Q*_*I*_ on *u* is neglected for reasons of efficiency. The other terms are derived analogously. The derivative of the combined distance measure (7) is therefore

(A.2) 𝒟′[u,φ]=∑k=1n∫ΩαkQXk  (X1k(  ·+u)−X0k)   ×∇X1k(  ·+u)φdx.

#### A.2. Regularization Term

We will continue by calculating the first variation of the regularization term *ℛ*_*g*_[*u*] defined in (8).

(A.3) ℛg′[u,φ]=ddεℛd[u+εφ]|ε=0=ddε12∫Ω||∇u(x)+ε∇φ(x)||2dx|ε=0=∫Ω∇u:∇φ  dx=∑i=1d∫Ω∇ui·∇φidx.

The divergence regularization term introduced in (9) is differentiated as follows:

(A.4) ℛd′[u,φ]=ddε12∫Ω(div⁡u+εdiv⁡φ)2  dx|ε=0=∫Ωdiv⁡udiv⁡φdx.

Therefore, the derivative of the full regularization functional *ℛ*[*u*] from (9) is

(A.5) ℛ′[u,φ]=μℛg′[u,φ]+νℛd′[u,φ]=∫Ωμ  ∇u:∇φ+νdiv⁡udiv⁡φdx.

*Statistical Prior.* Taking into account that the covariance operator *𝒞* is linear and self-adjoint and therefore this is also true for its pseudoinverse, it is easy to compute that the first variation of the statistical prior from (12) is given by

(A.6) 𝒫′[u,φ]=ddε𝒫(u+εφ)|ε=0=ddεγ2〈u+εφ−u¯,𝒞†[u+εφ−u¯]〉 +γ2〈u+εφ−u¯,σ−2(u+εφ−u¯)〉|ε=0=γ2〈u−u¯,𝒞†φ〉+γ2〈φ,𝒞†[u−u¯]〉 +γ2〈u−u¯,σ−2φ〉+γ2〈φ,σ−2(u−u¯)〉=∫Ωγ  𝒞†[u−u¯](x)·φ(x)dx +∫Ωγ  σ−2(u−u¯)(x)·φ(x)dx,

where 〈·, ·〉 = 〈·, ·〉_*L*^2^(*Ω*,ℝ^3^)_.

#### A.3. Strong Derivative

In order to derive the strong formulation, for the functional, we first apply integration by parts on the derivative of the regularization term (A.5). Assuming zero Neumann or Dirichlet boundary conditions, the boundary terms vanish and we get

(A.7) $$\begin{array}{c} {ℛ}^{\prime }\left[u,\varphi \right]=-\int_{\Omega }\mu \Delta u\cdot \varphi +\nu \nabla \mathrm{div}u\cdot \varphi dx. \end{array}$$

Now we can collect all the terms making up the weak derivative as follows:

(A.8) $$\begin{array}{c} {𝒥}^{\prime }\left[u,\varphi \right]={𝒟}^{\prime }\left[u,\varphi \right]+{ℛ}^{\prime }\left[u,\varphi \right]+{𝒫}^{\prime }\left[u,\varphi \right] \end{array}$$

from (A.2), (A.7), and (A.6) and apply the fundamental lemma of the calculus of variations to each component *φ*_*i*_ of *φ* which leads to the partial differential equation characterizing *u*:

(A.9) −μΔu−ν∇div⁡u+γ𝒞†[u−u¯] =−∑k=1nαiQXk(x)  (X1k(x+u(x))−X0k(x))    ×∇X1k(x+u(x)).

This is the well-known equation from linear elasticity with a forcing term on the right hand side and the additional term from the statistical deformation prior.
